# Microsurgical management of cranial dural arteriovenous fistulas: Treating the “Sheep in Wolf's clothing”? – Three decades of experience and lessons learned

**DOI:** 10.1016/j.bas.2025.105860

**Published:** 2025-10-26

**Authors:** Beate Kranawetter, Tammam Abboud, Fares Komboz, Anke Höllig, Hans Clusmann, Dorothee Mielke, Veit Rohde

**Affiliations:** a Department of Neurosurgery, University Medical Center Göttingen, Göttingen, Germany; b Department of Neurosurgery, University Hospital Augsburg, Augsburg, Germany; c Department of Neurosurgery, University Hospital Aachen, Aachen, Germany

**Keywords:** Cranial dural arteriovenous fistula, Microsurgery

## Abstract

**Introduction:**

Cranial dural arteriovenous fistulas (dAVFs) are rare vascular lesions and a growing number are treated with endovascular techniques. However, drawing on over three decades of clinical experience, we contend that microsurgery continues to play a crucial role in the management of these lesions.

**Research question:**

This study aims to highlight the safety and efficacy of microsurgical treatment for dAVFs, while providing practical insights into technical considerations and potential pitfalls.

**Materials and methods:**

Medical records, imaging studies, and surgical reports of patients diagnosed with a dAVF and treated with microsurgery between 1990 and 2025 were reviewed. We evaluated presenting symptoms, location, surgical strategy, surgical complications, occlusion status, and associated morbidity. Additionally, four representative cases were selected to illustrate technical aspects of the procedure and intraoperative challenges.

**Results:**

Overall, 82 dAVFs were treated with microsurgery. Mean patient age was 60.1 years (SD ± 9.9 years) with a male predominance (60/22). Hemorrhagic presentation was observed in 49 % (40/82) of patients. The most common locations were the tentorium (40 %, 33/82), superior sagittal/transverse sinus (20 %, 16/82), convexity (13 %, 11/82), and anterior cranial fossa (13 %, 11/82). Complete occlusion was achieved in 89 % (73/82) of cases after the initial surgery. Surgical complications occurred in 10 % of cases (8/82), with procedure-related morbidity in 4 % (3/82). After additional treatment, the overall occlusion rate increased to 96 % (79/82). There were no surgery- or hemorrhage-related deaths, resulting in a mortality rate of 0 %.

**Discussion and conclusion:**

Microsurgery is an immediate, safe, and effective treatment option for dAVFs.

## Highlights

• Complete occlusion of the dural fistula was achieved in 89 % (73/82) after the first treatment with microsurgery.
• After the second treatment complete occlusion rate increased to 96 % (79/82).
• Surgical complications occurred in 10 % of patients, with a permanent procedure-related morbidity of 4 %.

## 1 Introduction

Dural arteriovenous fistulas (dAVFs) are rare vascular pathologies characterized by pathological shunting between meningeal arteries and a dural venous sinus or a cortical vein. Characteristic locations include the tentorium, cavernous sinus (CS), transverse-sigmoid sinus (TSS), superior sagittal sinus (SSS), and the anterior cranial fossa (Zipfel et al., 2009). Clinical presentation is highly variable and is largely determined by the location and the hemodynamic characteristics, which are reflected in multiple classification systems (Zipfel et al., 2009; Borden et al., 1995; Cognard et al., 1995). The risk of severe nonhemorrhagic neurological deficits (NHND) and intracranial hemorrhage (ICH) is considered low (0.0 %–0.6 %) in dAVFs without cortical venous drainage (CVD) (Davies et al., 1997; Gross and Du, 2012; Satomi et al., 2002; Shah et al., 2012). In contrast, dAVFs with CVD are associated with a significantly higher risk, with reported annual event rates ranging from 7.4 % to 19.0 %, and an annual mortality rate of 3.8 % (Zipfel et al., 2009; Strom et al., 2009). Accordingly, complete disconnection of the arteriovenous (AV) shunt is essential in the management of high-grade fistulas. Treatment modalities include microsurgery, endovascular embolization, stereotactic radiosurgery, or a combination of these approaches. Over the recent decades, endovascular treatment (EVT) has become increasingly favored in the management of cerebrovascular pathologies, including dAVFs, while contemporary series evaluating microsurgery as a primary treatment method have become scarce (Al- et al., 2015; van et al., 2004; Gross and Du, 2013; Kakarla et al., 2007; Liu et al., 2009). Drawing on over 3 decades of clinical experience, this study aims to highlight the safety and efficacy of microsurgery in the management of dAVFs, while also outlining technical aspects of the procedure and emphasizing potential intraoperative challenges.

## 2 Patients and methods

### 2.1 Study design

This study presents a consecutive series of patients diagnosed with a cranial dAVF who underwent microsurgical treatment between January 1990 and March 2025. Clinical data were extracted from medical records and imaging studies, including digital subtraction angiography (DSA), magnetic resonance imaging (MRI)/MR angiography (MRA), as well as computed tomography (CT)/CT angiography (CTA). The study complied with the Declaration of Helsinki and received approval from the institutional ethics committee (study ID: 13/10/23).

#### 2.1.1 Diagnosis

Asymptomatic patients and those presenting with mild symptoms typically underwent initial evaluation with MRI/MRA and were subsequently referred by their general practitioner or neurologist for further assessment. In contrast, patients presenting with severe NHND or acute neurological deterioration due to ICH typically presented in an emergency setting and initially underwent CT/CTA imaging. When imaging indicated the presence of a dAVF, all patients underwent six-vessel DSA for comprehensive assessment of the lesion's angioarchitecture and hemodynamics. After evaluation, treatment decision was made in an interdisciplinary neurovascular board, compromising neurosurgeons, interventional neuroradiologists, and neurologists. In cases of acute clinical deterioration, the most appropriate treatment approach was determined by the on-call neurosurgeon and neuroradiologist.

#### 2.1.2 Surgical technique

CTA images were used for neuronavigation to assure a precise placement of the craniotomy above either the arterialized cortical vein or the arterialized part of the venous sinus. Caution was exercised during the exposure, as enlarged transosseous and dural feeders may lead to increased bleeding. For defining the operative strategy, dural fistulas can be categorized into sinus-type and non-sinus-type lesions. Sinus-type fistulas are characterized by multiple small arterial feeders that arterialize a portion of a venous sinus. The operative strategy for these lesions involved exposure of the arterialized sinus segment, incision of the extradural sinus wall, and packing with oxidized cellulose. Non-sinus-type fistulas, by contrast, consist of a well-defined AV-shunt between the feeding arteries and a cortical draining vein. The disconnection of the AV-shunt was performed after opening the dura by coagulating or clipping of the arterialized leptomeningeal vein as close to the intradural shunt point as possible. Feeding arteries visible during the exposure were also coagulated, but no attempt was made in both sinus-type and non-sinus-type fistulas to interrupt the feeders completely. After disconnection, intraoperative indocyanine green (ICG) video-angiography and micro-Doppler sonography were used to confirm complete obliteration of the fistula. Although these surgical principles apply for most fistulas, cranial approaches and intraoperative strategies vary depending on location and angioarchitecture.

### 2.2 Follow-up

All patients underwent CT/CTA imaging approximately 4 h postoperatively to assess for immediate complications. DSA was performed either within the following days after the surgery or at a follow-up visit after neurological recovery. If postoperative DSA confirmed complete occlusion of the fistula, the institutional protocol recommended annual clinical evaluations and follow-up imaging with MRA or CTA for a minimum of three years. Repeat angiography was reserved for cases in which follow-up imaging revealed an arterialized vein or sinus segment, or if expected clinical improvement was not observed.

## 3 Results

82 patients with a dAVF were treated with microsurgery between 1990 and 2025. Patient characteristics are summarized in Table 1. Mean patient age was 60.1 years (SD ± 9.9) and 73 % (60/82) of patients were male. Approximately, half of the patients (49 %, 40/82) presented with intracranial hemorrhage (ICH). Most common neurological symptoms at diagnosis were headaches 23 % (19/82), followed by seizures 11 % (9/82), and gait disturbances 10 % (8/82). However, clinical presentation was highly variable and also included nausea, aphasia, visual deficits, chemosis, impaired memory, and others. Twelve (14 %) patients were diagnosed incidentally. Excluding the two cavernous sinus fistulas (CSFs), 19 % (15/80) were classified as Borden type II and 81 % (65/80) as Borden type III. Most frequent anatomical locations were the tentorium 40 % (33/82), superior sagittal/transverse sinus 20 % (16/82), convexity 13 % (11/82) and anterior cranial fossa 13 % (11/82). Detailed information regarding fistula location, surgical approach, technique, and complications is summarized in Table 2.

**Table 1:** Summary of patient demographics and fistula characteristics *(n = 82).* Values are presented as mean ± SD or n (%).

	n = 82, n (%)
**Age (mean ± SD)**	60.1 ± 9.9
Min	35
Max	83
Sex (m:f)	60:22
**Location**	
Tentorial	33 (41)
SSS/TSS	16 (20)
Convexity	11 (13)
Anterior fossa	11 (13)
Foramen magnum	7 (9)
CS	2 (2)
Temporal	2 (2)
**Borden classificatio**n^a^	
II	13 (16)
III	67 (84)
**Hemorrhage**	
Yes	40 (49)
No	42 (51)
**Symptoms**	
Headache	19 (23)
Seizures	9 (11)
Gait disturbances/ataxia	8 (10)
Paresis/Tetraparesis	7 (9)
Aphasia	5 (6)
Impaired consciousness	5 (6)
Nausea	4 (5)
Visual impairment	3 (4)
Others	10 (12)
Incidental finding	12 (14)

CS; cavernous sinus, SSS; superior sagittal sinus, TSS; transverse-sigmoid sinus.

a The Borden Classification does not apply to cavernous sinus fistulas; therefore, these were excluded in this category, and all percentages are based on n = 80.

**Table 2:** Overview of fistula characteristics, surgical strategies, and postoperative complications.

ID	Type	Location	Approach	Technique	Surgical complications	New neurological deficit	Occlusion	Retreatment
**1**	NST	Tentorial	OC	SD	–	–	Residual	MS + EVT
**2**	NST	Tentorial	OC	SD	–	–	CO	–
**3**	NST	Tentorial	OC	SD	–	–	CO	–
**4**	NST	Tentorial	OC	SD	–	–	CO	–
**5**	NST	Tentorial	OC/SO	SD	–	–	CO	–
**6**	NST	Tentorial	OC/SO	SD	–	–	CO	–
**7**	NST	Tentorial	OC/SO	SD	–	–	CO	–
**8**	NST	Tentorial	OC/SO	SD	–	–	Residual	MS
**9**	NST	Tentorial	RS	SD	–	–	CO	–
**10**	NST	Tentorial	RS	SD	–	–	CO	–
**11**	NST	Tentorial	RS	SD	–	–	CO	–
**12**	NST	Tentorial	RS	SD	–	–	CO	–
**13**	NST	Tentorial	RS	SD	–	–	CO	–
**14**	NST	Tentorial	RS	SD	–	–	CO	–
**15**	NST	Tentorial	RS	SD	–	–	CO	–
**16**	NST	Tentorial	RS	SD	SSI	–	CO	–
**17**	NST	Tentorial	RS	SD	–	–	CO	–
**18**	NST	Tentorial	RS	SD	–	–	CO	–
**19**	NST	Tentorial	RS	SD	SSI	–	CO	–
**20**	NST	Tentorial	SO	SD	–	–	CO	–
**21**	NST	Tentorial	SO	SD	–	–	CO	–
**22**	NST	Tentorial	SO	SD	–	–	CO	–
**23**	NST	Tentorial	SO	SD	–	–	CO	–
**24**	NST	Tentorial	SO	SD	Infarction	–	Residual	None
**25**	NST	Tentorial	SO	SD	–	–	CO	–
**26**	NST	Tentorial	SO	SD	–	–	CO	–
**27**	NST	Tentorial	SO	SD	–	–	CO	–
**28**	NST	Tentorial	SO	SD	–	–	CO	–
**29**	NST	Tentorial	SO	SD	–	–	CO	–
**30**	NST	Tentorial	SO	SD	Rebleeding	–	CO	–
**31**	NST	Tentorial	SO	SD	–	–	CO	–
**32**	NST	Tentorial	SO	SD	–	–	CO	–
**33**	NST	Tentorial	ST	SD	–	–	CO	–
**34**	NST	Ethmoidal	Frontal	SD	Pneumocephalus	–	CO	
**35**	NST	Ethmoidal	Frontal	SD	–	Hyposmia	CO	
**36**	NST	Ethmoidal	Frontal	SD	–	–	Residual	MS
**37**	NST	Ethmoidal	IH	SD	–	–	CO	
**38**	NST	Ethmoidal	IH	SD	–	–	CO	
**39**	NST	Ethmoidal	IH	SD	–	–	CO	
**40**	NST	Ethmoidal	IH	SD	–	–	CO	
**41**	NST	Ethmoidal	IH	SD	–	–	CO	
**42**	NST	Ethmoidal	IH	SD	–	–	CO	
**43**	NST	Ethmoidal	IH	SD	–	–	CO	
**44**	NST	Ethmoidal	IH	SD	SSI	–	Residual	MS
**45**	NST	Convexity	Frontal	SD	–	–	CO	–
**46**	NST	Convexity	OC	SD	Rebleeding	Hemianopsia	CO	–
**47**	NST	Convexity	OC	SD	–	–	Residual	MS
**48**	NST	Convexity	Parietal	SD	–	–	CO	–
**49**	NST	Convexity	Parietal	SD	–	–	CO	–
**50**	NST	Convexity	Parietal	SD	–	Hemiparesis	CO	–
**51**	NST	Convexity	Parietal	SD	–	–	CO	–
**52**	NST	Convexity	Parietal	SD	–	–	CO	–
**53**	NST	Convexity	Parietal	SD	–	–	Residual	MS
**54**	NST	Convexity	Temporal	SD	–	–	CO	–
**55**	NST	Convexity	Temporal	SD	–	–	CO	–
**56**	NST	FM	FME	SD	–	–	CO	–
**57**	NST	FM	FME	SD	–	–	CO	–
**58**	NST	FM	FME	SD	–	–	CO	–
**59**	NST	FM	SO	SD	–	–	CO	–
**60**	NST	FM	SO	SD	–	–	CO	–
**61**	NST	FM	SO	SD	–	–	CO	–
**62**	NST	FM	SO	SD	–	–	CO	–
**63**	NST	SSS/TS	RS	SD	–	–	CO	–
**64**	NST	SSS/TS	SO	SD	–	–	CO	–
**65**	NST	SSS/TS	SO	SD	–	–	CO	–
**66**	NST	Temporal	Subtemporal	SD	–	N. IV palsy	CO	–
**67**	NST	Temporal	Pterional	SD	–	–	CO	–

**68**	ST	SSS/TS	OC/SO	Transection	Rebleeding	–	CO	–
**69**	ST	SSS/TS	OC/SO	Packing	–	–	CO	–
**70**	ST	SSS/TS	OC/SO	Packing	–	–	CO	–
**71**	ST	SSS/TS	OC/SO	Packing	–	Hemianopsia	CO	–
**72**	ST	SSS/TS	OC/SO	Packing	–	–	Residual	MS
**73**	ST	SSS/TS	Parieto-OC	Packing	–	–	CO	–
**74**	ST	CS	Pterional	Packing	–	–	CO	–
**75**	ST	CS	Pterional	Packing	–	–	Residual	MS
**76**	ST	SSS/TS	RS	Packing	–	–	CO	–
**77**	ST	SSS/TS	RS	Packing	–	–	CO	–
**78**	ST	SSS/TS	RS	Packing	–	–	CO	–
**79**	ST	SSS/TS	SO	Packing	–	–	CO	–
**80**	ST	SSS/TS	SO	Packing	–	–	CO	–
**81**	ST	SSS/TS	SO	Transection	–	–	CO	–
**82**	ST	SSS/TS	SO	Packing	–	–	CO	–

EVT; endovascular treatment, CO; complete occlusion, FM; foramen magnum, FME; foramen magnum extension, IH; interhemispheric, MS; microsurgery, NST; non-sinus-type, OC; occipital, RS, retrosigmoid, SD; selective disconnection, SO; suboccipital, SSI; surgical side infection, SSS; superior sagittal sinus, ST; sinus-type, TS; transverse sinus.

### 3.1 Occlusion rate and morbidity

Postoperative angiographic evaluation was performed in all patients revealing complete occlusion in 89 % (73/82) following the initial surgical intervention. Of the nine patients with residual AV-shunting, eight underwent repeat microsurgical treatment. One of them was additionally retreated with EVT. One patient did not undergo further intervention due to poor clinical status. This patient had experienced an infratentorial hemorrhage and presented with a Glasgow Coma Scale (GSC) score of 3 preoperatively, thus further treatment was not pursued. Among the eight patients who underwent retreatment, complete occlusion was achieved in six cases, resulting in a complete occlusion rate of 96 % (79/82) after the second treatment. Importantly, all incompletely occluded lesions demonstrated a favorable change in angioarchitecture, being downgraded from Borden type III to Borden type I.

None of the patients died due to the surgery or as a consequence of the initial bleeding. Surgical complications occurred in 10 % of cases (8/82) and included three surgical site infections (SSI), three cases of rebleeding, one cerebral infarction, and one case of pneumocephalus requiring surgical intervention. New neurological deficits were reported in five patients including hyposmia in one patient with an ethmoidal fistula, hemianopsia in two patients with fistulas involving the occipital region, hemiparesis in a patient with a parietal fistula, and trochlear nerve palsy in a patient with a tentorial fistula treated via a subtemporal approach. Notably, the trochlear nerve palsy and hemiparesis were transient, leading to a permanent procedure-related morbidity of 4 % (3/82). One patient was lost to follow-up. Among the remaining cohort, the mean duration of follow-up was 2.7 (SD ± 2.3) years. Neurological symptoms improved in 79 % (65/82) of patients. Over the follow-up period, a reperfusion of the initially occluded fistulas was observed in 4 % (3/82). Interestingly, the incompletely occluded fistulas remained stable.

#### 3.1.1 Surgical cases

##### 3.1.1.1 Case 1 – cavernous sinus fistula

**Fig. 1:**
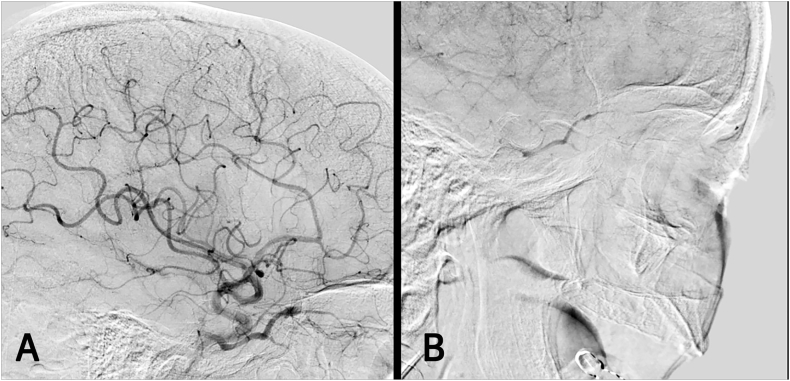
**(A)** Preoperative angiogram demonstrating a cavernous sinus fistula (CSF) with early filling of the ophthalmic vein. **(B)** Digital subtraction angiography (DSA) performed three months postoperatively reveals recurrent arteriovenous shunting.

A 59-year-old female presented with chemosis, exophthalmos, and oculomotor palsy on the right eye. DSA confirmed the presence of a right-sided CSF (Fig. 1A). Surgical access to the CS was achieved via the anteromedial triangle, and the sinus was packed with oxidized cellulose. Three months postoperatively, the patient experienced a recurrence of her symptoms and follow-up angiography revealed refilling of the fistula (Fig. 1B). Inspection of the previously opened CS demonstrated an absence of the oxidized cellulose, likely due to its complete resorption. Consequently, the sinus was repacked using a combination of oxidized cellulose, cottonoids, and fibrin glue. Additionally, the sinus opening was sealed with TachoSil. Following the second surgery, the patient's symptoms resolved completely. Postoperative DSA confirmed complete occlusion of the fistula.

##### 3.1.1.2 Case 2 – complex Borden type III fistula located at the superior Saggital sinus

**Fig. 2:**
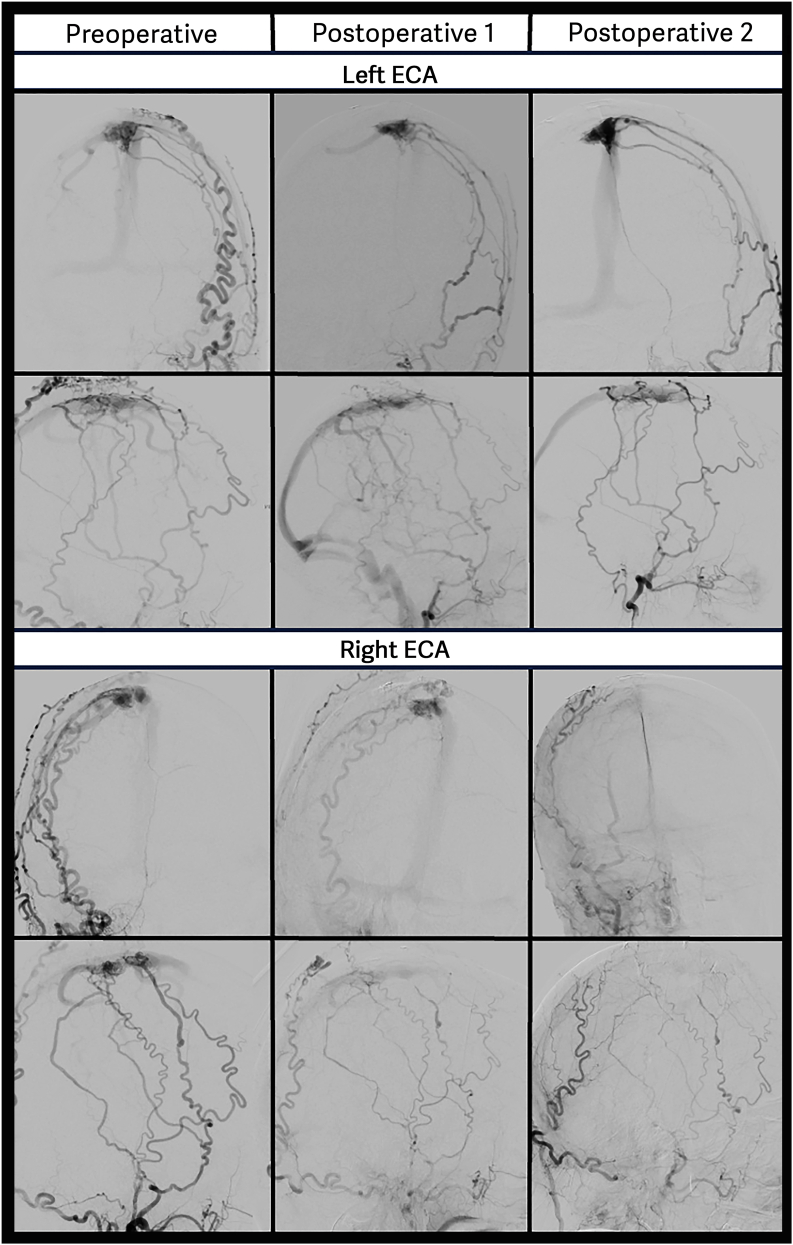
The upper portion of the figure depicts preoperative and postoperative DSA of the left external carotid artery (ECA) in anteroposterior (A.P.) and lateral projections. The lower portion displays corresponding views of the right ECA. After the first surgery, the lateral and A.P. views of the left ECA demonstrate early filling of a cortical vein located dorsally to the initial high-grade shunt, a finding not evident in the preoperative imaging. As a result, a second surgery was performed. The final DSA shows complete occlusion of the high-grade fistula, but persistent direct shunting into the superior sagittal sinus (Borden type I), as expected.

Papilledema was diagnosed by the ophthalmologisFt in a 58-year-old patient, prompting further investigation. MRI revealed a dAVF located at the SSS. DSA demonstrated a complex AV-shunt consisting of high- and low-grade fistulous components, with multiple feeding arteries from both the internal and external carotid artery. Due to the several arterial feeders directly shunting into the SSS surgical strategy was to occlude the high-grade part of the lesion down-grading it to a benign Borden type I lesion. A navigated craniotomy was performed above the fistulous point. The initial segment of the arterialized draining vein was visualized, coagulated, and transected. Additionally, the wall of the SSS appeared thickened, and several small arterial feeders were detected and coagulated, which subsequently led to a reduction in the size of the sinus wall. Postoperative angiography showed an occlusion of the targeted dAVF. However, likely due to the loss of anterior arterial inflow into the dural layers, a second leptomeningeal draining vein, located posteriorly to the craniotomy, became apparent (Fig. 2). A second surgery was performed, and the trepanation was extended posteriorly. The dura was opened, the arterialized vein was clearly visible and was confirmed with micro-Doppler. The vessel was coagulated and transected, and both micro-Doppler and ICG angiography confirmed complete cessation of flow. Final postoperative DSA showed successful down-grading of the fistula to Borden type I (Fig. 2). The patient has been followed up for 4 years and the fistula has remained stable.

##### 3.1.1.3 Case 3 –ethmoidal fistula with a large, thrombosed venous aneurysm

**Fig. 3:**
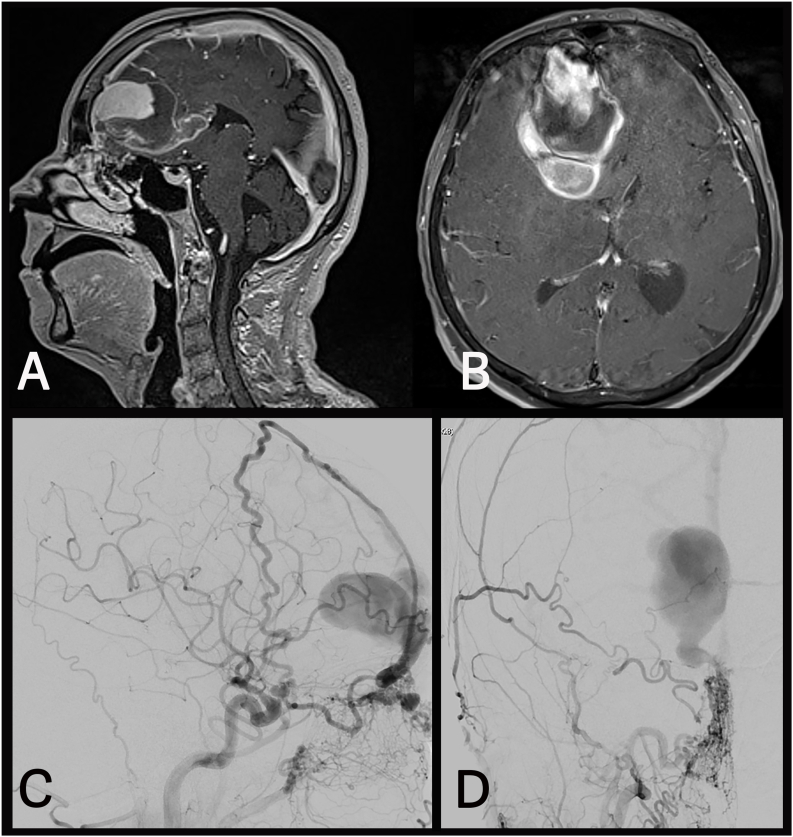
**(A)** and **(B)** show preoperative MR images of the ethmoidal fistula with a large venous aneurysm. The initially suspected intracerebral hemorrhage (ICH) posteriorly to the aneurysm, was intraoperatively identified as a thrombosed component of the venous outpouching. **(C)** and **(D)** show preoperative lateral and an anteroposterior (A.P.) projections of the fistula with feeders originating from the ethmoidal arteries.

This 73-year-old patient had experienced severe headaches, nausea, and vomiting. MRI/MRA revealed an anterior fossa dAVF and associated ICH (Fig. 3A–B). DSA confirmed an ethmoidal fistula with a giant aneurysm of the draining vein (Fig. 3C–D). A right-sided frontal craniotomy was performed. Upon deeper dissection, it became apparent that the MRI had indicated a thrombosed segment of the aneurysm, not a hemorrhage as initially suspected. Therefore, reaching the frontal base was extremely challenging and the trepanation was extended to the left. Gradual incision of the falx led to a massive bleeding, which could only be controlled by tamponade with cottonoids. Consequently, access to the fistula from the left was no longer feasible, necessitating an extension of the craniotomy to the right. Using neuronavigation, the proximal segment of the draining vein was identified and ligated with a clip, which stopped the bleeding. Inspection of the interhemispheric fissure revealed that the wall of the venous aneurysm was adherent to the falx, leading to its inadvertent opening. Due to the mass effect the large, thrombosed portion of the aneurysm was resected. Arterial bleeding recurred, necessitating the placement of second clip, which led to complete hemostasis. Postoperative DSA confirmed complete occlusion of fistula. However, a second dAVF, supplied by falcine meningeal arteries with cortical drainage, was identified and a second surgery was planned. Using neuronavigation, the fistula and the proximal segment of the arterialized draining vein were located at the anterior margin of the right-sided craniotomy. The vein was coagulated and divided just distal to its exit from the sinu-falcine dura. Intraoperative micro-Doppler showed cessation of flow, and the postoperative DSA confirmed complete occlusion.

##### 3.1.1.4 Case 4 – foramen magnum fistula

**Fig. 4:**
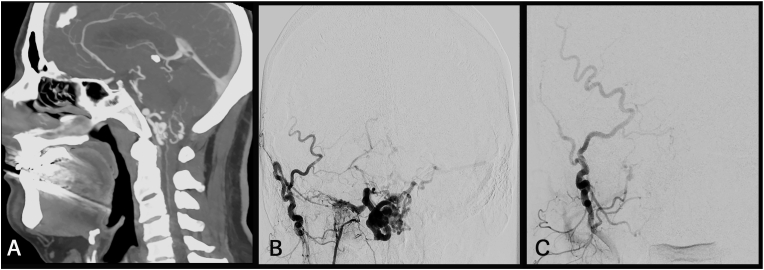
**(A)** Preoperative CTA displaying a foramen magnum fistula with enlarged veins within the posterior fossa. **(B)** Preoperative angiogram demonstrating the AV-fistula with feeders from the external carotid artery (ECA) in anterior posterior (A.P.) projection. **(C)** Postoperative digital subtraction angiography (DSA) depicting complete occlusion of the fistula.

The patient experienced a transient ischemic attack, and CTA revealed dilated veins surrounding the medulla (Fig. 4A). Subsequent DSA demonstrated a dAVF located at the foramen magnum in close proximity to the right jugular foramen (Fig. 4B). The arterialized draining veins were mainly located in the posterior cranial fossa, with venous drainage into the right basal vein of Rosenthal and the straight sinus. The patient underwent surgery in a sitting position via a suboccipital approach, or more specific a slightly right-sided enlargement of the foramen magnum. An ectatic draining vein was identified and traced anterolaterally to its origin near the jugular foramen. ICG and micro-Doppler confirmed the pathology. Given the depth and limited size of the intradural working corridor, a bayonet clip was applied to occlude the AV-fistula. Cessation of flow in the arterialized draining vein was verified using ICG and micro-Doppler, and complete obliteration was confirmed on postoperative angiography (Fig. 4C).

## 4 Discussion

With an occlusion rate of 89 % following initial surgery and a permanent surgery-related morbidity of 4 %, our findings demonstrate that microsurgery is an effective and safe treatment option. Despite their often complex and intimidating angioarchitecture, once properly understood, microsurgery is a straight-forward and ultimate treatment option in most cases. In this context, AVF surgery may be viewed as addressing the “sheep in wolf's clothing”. However, beyond highlighting the overall effectiveness of microsurgical disconnection in standard cases, we also sought to present real-world examples that illustrate the practical realities of surgical decision-making, and the intraoperative challenges encountered in more complex lesions.

Although microsurgery is a highly effective treatment option, recent advances in EVT have established it as a valid alternative for certain types of dAVFs. A prominent example are CSFs, where EVT now serves as the first-line treatment. A comprehensive review of 22 studies including 1043 patients with a CSF found that transvenous approaches achieved complete occlusion in 80 % of cases, with a complication rate of 8 % (Alexandre et al., 2022). Although surgical series reported occlusion rates from 31 % to 100 % (Isamat et al., 1986; Ringer et al., 2005; Ellis et al., 2012; Day and Fukushima, 1997; Tu et al., 1997) EVT has proven to be technically superior since it spares surrounding cranial nerves and enables precise occlusion of the fistulous point while preserving normal venous outflow. Furthermore, packing can be insufficient, as illustrated in the presented case. The reduced invasiveness of EVT has not only led to its establishment as the gold standard for treating fistulas of the CS but also sinus-type fistulas in general (Xu et al., 2018). Studies evaluating EVT for fistulas of the transverse-sigmoid junction and SSS reported complete occlusion rates between 67 % and 95 %, with associated complication rates ranging from 0 % to 7.8 % (Tsukada et al., 2023; Xu et al., 2019; Piechowiak et al., 2017; Chandra et al., 2014; Kurabe et al., 2021).

Regarding non-sinus-type fistulas, there exists a broad consensus that cases presenting with aggressive clinical features require urgent intervention to mitigate the risk of bleeding and progressive NHND. Our experience, along with that of others (Al- et al., 2015; Gross and Du, 2013; Kakarla et al., 2007; Liu et al., 2009), has shown that selective disconnection via microsurgery results in excellent occlusion rates, low complication rates, and robust protection against future hemorrhage. However, at the same time, EVT has also demonstrated favorable occlusion rates with an acceptable safety profile in high-grade dAVFs. A recent study reported a complete occlusion rate of 80 %, with procedural complications occurring in 20 % (Mendez-Ruiz et al., 2022). While these findings underscore the growing role of EVT as a viable treatment option for dAVFs with CVD, stratification by location suggests that microsurgery may provide superior outcomes. Ethmoidal dAVFs exemplify cases where microsurgery may be preferred, as catheter access is technically challenging and carries the risk of occluding the ophthalmic artery. A meta-analysis by Giannopoulos et al. (2019) reported a 100 % obliteration rate with microsurgery versus 47 % with EVT. This was supported by a more recent meta-analysis by Berke et al. (2024), showing higher rates of complete occlusion with microsurgery (89 % vs. 70 %) and comparable procedure-related complication rates between the two modalities (10 % for surgery vs. 13 % for EVT). In our cohort, ethmoidal fistulas were completely occluded in 82 % (9/11), with a procedure-related complication rate of 18 % (2/11). The presented surgical case underscores the potential risks associated with markedly enlarged draining veins and venous varices which are common in ethmoidal fistulas. Notably, preoperative imaging should be interpreted with caution, as thrombosed varices may lack contrast filling on angiography, leading to underestimation of their true size.

Another distinct and rare subtype of dAVFs, are those located at the foramen magnum. Due to their rarity, only a limited number of case series have been published on the microsurgical treatment of these lesions (Xiao et al., 2023; Takami et al., 2005; Guo et al., 2010). A recent systematic review and meta-analysis identified six case series and 21 case reports including 56 foramen magnum dAVFs. The majority were treated with EVT (75 %, 42/56), while 23 % (13/56) were managed surgically. The occlusion rates were 88 % for EVT and 100 % for microsurgical treatment, with complication rates of 7 % and 0 %, respectively (Hiramatsu et al., 2025). In our series, all foramen magnum fistulas (n=7) were completely occluded without procedure-related complications or morbidity. As demonstrated in the presented case, despite their complex angioarchitecture, surgical treatment is often straightforward when the shunt point is accurately identified.

Tentorial fistulas occupy an intermediate position. A comprehensive review found that endovascular therapy alone achieved a complete occlusion rate of 71 %, combined endovascular and surgical treatments reached 84 %, and surgery alone 81 % (Cannizzaro et al., 2015). The advent of newer liquid embolic agents such as Onyx, Squid, and PHIL has significantly advanced EVT of these lesions. A recent meta-analysis including 214 patients demonstrated a complete occlusion rate of 91 % with these agents (Günkan et al., 2024). Similarly, Onyx-based EVT yielded an occlusion rate of 88 % in tentorial dAVFs (Voldřich et al., 2021). Another large single-center series including 275 cases reported a 93 % occlusion rate and a 13 % complication rate with EVT (Su et al., 2025). In our series, tentorial fistulas were managed with microsurgical disconnection without prior embolization, achieving a complete occlusion rate of 91 % (3/33). These results align with findings by Lawton et al. (2008), who reported a 94 % (29/31) occlusion rate; however, in contrast to our series, all patients underwent preoperative embolization. While both EVT and surgery have shown comparable efficacy, particularly with the introduction of newer embolic agents, the long-term durability of EVT, especially when used as a stand-alone treatment, remains insufficiently studied.

## 5 Conclusion and institutional decision process

With EVT being the less invasive therapeutic option and its continued advancements, our institutional treatment paradigm has evolved over the past two decades. Sinus-type fistulas are now managed with EVT, while microsurgery is used as a second-line treatment for residual lesions. In contrast, our experience with non-sinus-type fistulas has demonstrated that selective microsurgical disconnection of CVD achieves high occlusion rates, low procedure-related morbidity, effective prevention of rebleeding, and a low recurrence rate. As a result, microsurgery remains the first-line treatment for ethmoidal, foramen magnum, and convexity dAVFs with CVD. This approach may not be universally applied, but tentorial fistulas are also treated primarily with microsurgery in our center. Nonetheless, we acknowledge that occlusion rates achieved with newer liquid embolic agents are now comparable to those of microsurgery. Accordingly, in high-volume centers with significant endovascular expertise, EVT may reasonably be considered as primary treatment modality in selected cases. Another factor influencing our treatment strategy is the clinical presentation. In our series, approximately half of the lesions were associated with ICH or/and subarachnoid hemorrhage (SAH), often prompting immediate intervention. For ruptured supratentorial lesions immediate surgery is typically performed without delay. For infratentorial lesions, timing of surgery depends on both hemorrhage volume and clinical status. If the bleeding causes significant mass effect, immediate surgery is performed. However, if the intraparenchymal part of the bleeding does not cause mass effect, it may be beneficial to delay surgery for 1–2 weeks to allow for resolution of edema and SAH in an already limited operative corridor. However, delaying the surgery carries the risk of rebleeding, which must be carefully weighed in the decision-making process.

## Consent to participate

Informed consent was not necessary since this is a retrospective study.

## Disclosure

The authors report no conflict of interest concerning the materials or methods used in this study or the findings specified in this paper.

## Ethics approval

The study was approved by the local ethics committee (application number: 13/10/23).

## Authors contributions

VR designed and supervised the study. BK and DM collected clinical data, BK performed statistical analyses. BK, TA and VR drafted the manuscript. All authors critically revised the manuscript and approved its final version.

## Funding

The authors received no extramural funding or financial support for the research, authorship or publication of this article.

## Declaration of competing interest

The authors declare that they have no known competing financial interests or personal relationships that could have appeared to influence the work reported in this paper.
